# Supplementary material for: Enhancing Self-Esteem and Body Image of Breast Cancer Women through Interventions: A Systematic Review
Source: Int J Environ Res Public Health. 2021 Feb 9;18(4):1640. doi: 10.3390/ijerph18041640 (PMC7914971; doi:10.3390/ijerph18041640)
Supplement: Supplementary file 1 [file ijerph-18-01640-s001.pdf]

Supplementary materials 1

Table S1. List of excluded studies and reasons for exclusion (n=29)

N.	Reference	Reason for exclusion
**1**	Boing, L.; do Bem Fretta, T.; de Carvalho Souza Vieira, M.; Pereira, G.S.; Moratelli, J.; Sperandio, F.F.; Bergmann, A.; Baptista, F.; Dias, M.; de Azevedo Guimarães, A.C. Pilates and dance to patients with breast cancer undergoing treatment: study protocol for a randomized clinical trial–MoveMama study. *Trials*, **2020**, *21(1)*, 1-17.	No applied intervention identified (Study protocol)
**2**	Franco, C.; Amutio, A.; Mañas, I.; Sánchez-Sánchez, L. C.; Mateos-Pérez, E. Improving psychosocial functioning in mastectomized women through a mindfulness-based program: Flow meditation. *Int J Stress Manage*, **2020**, *27(1)*, 74-81.	Body image not assessed
**3**	Esplen, M. J.; Wong, J.; Warner, E.; Toner, B. Restoring body image after cancer (ReBIC): results of a randomized controlled trial. *J Clin Oncol*, **2018**, *36(8)*, 749-756.	Self-esteem not assessed
**4**	Di Mattei, V. E.; Carnelli, L.; Taranto, P.; Bernardi, M.; Brombin, C.; Cugnata, F.; ... Candiani, M. "Health in the Mirror": An Unconventional Approach to Unmet Psychological Needs in Oncology. *Front Psychol.*, **2017**, *8(*1633), 1-6	No participants with breast cancer only / Results were not separately
**5**	Napoles, T. M.; Guerra, C.; Orenstein, F.; Luce, J. A.; Merritt, S.; Burke, N. J. Healing art: Breast cancer survivor experiences with nipple-areola tattoo procedures during breast reconstruction. Proceedings of the San Antonio Breast Cancer Symposium, San Antonio, Texas, USA, *Cancer Res*, **2017**, *78(4)*, P6-12-19	No applied intervention identified
**6**	Landry, S.; Chasles, G.; Pointreau, Y.; Bourgeois, H.; Boyas, S. Influence of an adapted physical activity program on self-esteem and quality of life of breast cancer patients after mastectomy. *Oncology*, **2018**, *95*(3), 188-191.	Body image not assessed
**7**	Marafante, G.; Ferri, C.; Giuffredi, I.; Bidin, L. Image and self-esteem: a photo-therapy program to improve body image, increase self-awareness and the expression of emotions in breast cancer patients. A pilot study. Proceedings of the 19th National Congress of Medical Oncology, Rome, Italy, *Ann Oncol*, **2017,** *28*, R16.	Conference Abstract / Full-text not available
**8**	Pike, C.M.; Firriolo, J.M.; Ontiveros, N.C.; Kuchibhotla, S.P.; Oppel, O.K.; Monoxelos, L.C.; ...; Labow, B.I. (2017). A nonsurgical approach to adolescent breast asymmetry using external prostheses. *J Adoles Health*, **2017**, *61(2)*, 240-245.	No participants with breast cancer
**9**	Volders, J.H., Negenborn, V.L., Haloua, M.H., Krekel, N.M., Jóźwiak, K., Meijer, S., & M. van den Tol, P. Cosmetic outcome and quality of life are inextricably linked in breast-conserving therapy. *J Surg Oncol*, **2017**, *115*(8), 941-948.	Body image and self-esteem not assessed / No applied intervention identified
**10**	Prates, A.C.L.; Freitas-Junior, R.; Prates, M.F.O.; Veloso, M. D.F.; Barros, N.D.M. Influence of body image in women undergoing treatment for breast cancer. *Rev Bras Ginecol Obstet*, **2017**, *39*(4), 175-183.	No applied intervention identified
**11**	Quintard, B.; Lakdja, F. Assessing the effect of beauty treatments on psychological distress, body image, and coping: a longitudinal study of patients undergoing surgical procedures for breast cancer. *Psychooncology*, **2008**, *17(10)*, 1032-1038.	Self-esteem not assessed

**12**	McGarvey, E.L.; Baum, L. D.; Pinkerton, R. C.; Rogers, L.M. Psychological sequelae and alopecia among women with cancer. *Cancer Pract.*, **2001**, *9(6)*, 283-289.	No applied intervention identified
**13**	Al-Ghazal, S.K.; Fallowfield, L.; Blamey, R.W. Does cosmetic outcome from treatment of primary breast cancer influence psychosocial morbidity? *Eur J Surg Oncol*, **1999**, *25*(6), 571-573.	No applied intervention identified
**14**	Izydorczyk, B., Kwapniewska, A., Lizinczyk, S., & Sitnik-Warchulska, K. Psychological resilience as a protective factor for the body image in post-mastectomy women with breast cancer. *Int J Environ Res Public Health*, **2018**, *15*(6), 1181.	Self-esteem not assessed / No applied intervention identified
**15**	Barel-Shoshani, Z.A.; Kreitler, S. Changes in self-perception following breast cancer as expressed in self-figure drawings: Present-past. *Art Psychother*, **2017**, *55*, 136-145.	Self-esteem not assessed
**16**	Sbitti, Y.; Kadiri, H.; Essaidi, I.; Fadoukhair, Z.; Kharmoun, S.; Slimani, K.; ... Errihani, H. Breast cancer treatment and sexual dysfunction: Moroccan women's perception. *BMC women's health*, **2011**, *11*(1), 1-5.	Self-esteem not assessed
**17**	Fobair, P., Stewart, S.L., Chang, S., D'Onofrio, C., Banks, P. J., & Bloom, J.R. Body image and sexual problems in young women with breast cancer. *Psychooncology*, **2006**, *15*(7), 579-594.	No applied intervention identified / Self-esteem not assessed as outcome
**18**	Pinto, B.M.; Trunzo, J.J. Body esteem and mood among sedentary and active breast cancer survivors. *Mayo Clin Proc*, **2004**, *79(2)*, 181-186.	Self-esteem not assessed / No applied intervention identified
**19**	Berterö, C.M. Affected self-respect and self-value: the impact of breast cancer treatment on self-esteem and QoL. *Psychooncology*, **2002**, *11*(4), 356-364.	Body image not assessed / No applied intervention identified
**20**	Goldov, N.B. The effects of individualized brief medical dance/movement therapy on body image in women with breast cancer. Doctoral Dissertation, Argosy University, Seattle, 2011.	Self-esteem not assessed / Doctoral Dissertation
**21**	Allen, J.L. *The effectiveness of group music psychotherapy in improving the self-concept of breast cancer survivors*. Doctoral Dissertation. Temple University. Philadelphia, Pennsylvania (USA). 2010.	Self-esteem not assessed / Doctoral Dissertation
**22**	Taylor, K.L.; Lamdan, R.M.; Siegel, J.E.; Shelby, R.; Moran-Klimi, K.; Hrywna, M. Psychological adjustment among African American breast cancer patients: One-year follow-up results of a randomized psychoeducational group intervention. *Health Psychol*. **2003**, *05;22(3)*, 316-323.	Body image and self-esteem not assessed / Not related with the topic
**23**	Antoni, M.H.; Lehman, J.M.; Kilbourn, K.M.; Boyers, A.E.; Culver, J.L.; Alferi, S.M. Cognitive-behavioral stress management intervention decreases the prevalence of depression and enhances benefit finding among women under treatment for early-stage breast cancer. *Health Psychol*, **2001**, *01;20(1)*, 20-32.	Body image and self-esteem not assessed / Not related with the topic
**24**	Helgeson, V.S.; Cohen, S. Social support and adjustment to cancer: Reconciling descriptive, correlational, and intervention research. *Health Psychol*. **1996**, *03;15(2)*, 135-148.	Systematic review / No body image and self-esteem as outcomes
**25**	Giese-Davis, J.; Koopman, C.; Butler, L.D.; Classen, C.; Cordova, M.; Fobair, P. Change in emotion-regulation strategy for women with metastatic breast cancer following supportive-expressive group therapy. *J Consult Clin Psychol* **2002**, *08;70(4)*, 916-925.	Body image and self-esteem not assessed / Not related with the topic

**26**	Lechner, S.C.; Carver, C.S.; Antoni, M.H.; Weaver, K.E.; Phillips, K.M. Curvilinear associations between benefit finding and psychosocial adjustment to breast cancer. *J Consult Clin Psychol* **2006**, *10;74(5)*, 828-840.	Self-esteem not assessed / No applied intervention identified / Observational study
**27**	Gilbar, O. Breast cancer: How do Israeli women cope? A cross-sectional sample. *Fam. Syst. Health* **2005**, *Jul;23(2)*, 161-171.	Body image and self-esteem not assessed / No applied intervention identified / Observational study
**28**	Tomich, P.L.; Helgeson, V.S. Is Finding Something Good in the Bad Always Good? Benefit Finding Among Women With Breast Cancer. *Health Psychol.* **2004**, *01;23(1)*, 16-23.	Body image and self-esteem not assessed / Observational study
**29**	Sellers, T.S. A model of collaborative healthcare in outpatient medical oncology. *Fam. Syst. Health* **2000**, *Apr;18(1)*, 19-33.	No applied intervention identified
